# COVID-19 and Psychosocial Support Services: Experiences of People Living with Enduring Mental Health Conditions

**DOI:** 10.1007/s10597-021-00871-0

**Published:** 2021-07-07

**Authors:** Anne Honey, Shifra Waks, Monique Hines, Helen Glover, Nicola Hancock, Debra Hamilton, Jennifer Smith-Merry

**Affiliations:** 1 Faculty of Medicine and Health, The University of Sydney, Sydney, Australia; 2 Enlightened Consultants, Brisbane, QLD Australia

**Keywords:** Mental health, COVID-19, Psychosocial support program, Lived experience perspectives, Community mental health support

## Abstract

This paper uses secondary analysis to understand how COVID-19 shaped people’s experiences with psychosocial support services in Australia. Data are drawn from questionnaires (n = 66) and semi-structured interviews (n = 62), conducted for a national service evaluation, with 121 people living with enduring mental health conditions and using psychosocial support services. Data relating to COVID-19 were inductively coded and analysed using constant comparative analysis. Most people’s experiences included tele-support. While some people described minimal disruption to their support, many reported reduced engagement. People’s wellbeing and engagement were influenced by: their location, living situation and pre-COVID lifestyles; physical health conditions; access to, comfort with, and support worker facilitation of technology; pre-COVID relationships with support workers; and communication from the organisation. The findings can help services prepare for future pandemics, adjust their services for a ‘COVID-normal’ world, and consider how learnings from COVID-19 could be incorporated into a flexible suite of service delivery options.

## Introduction

This study examines the impact of COVID-19 on people’s experiences of psychosocial support services. Psychosocial support refers to non-clinical services that assist people with enduring mental health conditions (EMHCs) to build skills to manage their mental health conditions, improve their relationships, and increase social and economic participation (Australian Government Department of Health, 2019).

COVID-19 and the resulting uncertainty and restrictions have had a negative impact on mental health in the community, with increased rates of psychological distress and mental health issues (Edwards et al., 2020; Fisher et al., 2020). People with EMHCs are likely to be particularly affected by the impacts of COVID-19. EMHCs (often referred to as serious or severe and persistent mental illness) are long lasting conditions which result in an impairment or restriction that can limit an individual’s ability to function, think clearly, maintain their physical health or manage their social and emotional welfare (Ruggeri et al., 2000; Schinnar et al., 1990). People with EMHCs are, as a group, thought to be vulnerable to COVID-19 due to: the higher prevalence of physical health conditions and lifestyle issues like smoking and substance use; lower access to physical health services; impact of medications on obesity; and lower overall life expectancy (Campion et al., 2020). Having an existing mental health condition is also likely to exacerbate the mental health impacts of the pandemic and the measures used to control it, for example, through increased stress and anxiety, loneliness and isolation, and the disrupted delivery of mental health and support services (Holmes et al., 2020). The need for uninterrupted access to mental health and support services for these people during the pandemic has been emphasised (World Health Organization, 2020).

Telehealth is one strategy proposed to enhance continuity of service delivery where barriers to face to face service delivery exist. While online psychosocial support services have not been examined, recent reviews have found high levels of feasibility and acceptability for tele-psychotherapy for individuals diagnosed with schizophrenia-spectrum disorders (Santesteban-Echarri et al., 2020) and that videoconferencing for psychological therapies was no less effective than face-to-face delivery for people with anxiety (Berryhill et al., 2019). Further a recent narrative review of therapeutic alliance in online psychotherapy concluded that therapeutic alliance, particularly from clients’ points of view, was high and comparable with face-to-face services (Berger, 2016). Similarly, evidence suggests that allied health services delivered via telehealth may be as effective as in-person services (Grogan-Johnson et al., 2010; Speyer et al., 2018) and they have repeatedly been found to be highly acceptable to service users (Crutchley & Campbell, 2010; Graham et al., 2020; Hines et al., 2019).

Apart from continued service delivery during a pandemic, additional benefits of telehealth are thought to include: increased access, particularly for clients in rural and remote areas or whose physical or mental health conditions prevent travel; cost-effectiveness; decreased travel time; convenience; ease of coordination; and the possibility of providing greater flexibility and choice (Dew et al., 2012; Johnson et al., 2021; Madigan et al., 2020; Nadeem et al., 2020). Experts have suggested that the necessity of adapting services during the pandemic could provide a long-term opportunity to improve service delivery and cost-effectiveness of mental health services (de Medeiros Carvalho et al., 2020; Stefana et al., 2020).

However, challenges have also been identified. These include the loss of in-person contact, confidentiality concerns, reduced non-verbal communication, technological problems, therapist fatigue, unsafe home environments, distraction, decreased depth of sessions, concerns around client safety, and reduced client commitment (Dausch et al., 2009; Downing & Harriott, 2020; McLaren et al., 1995; Roncero et al., 2020). Further, a significant proportion of people living with EMHCs lack consistent access to smartphones, data and reliable internet and may lack the ability to navigate online platforms (Johnson et al., 2021; Madigan et al., 2020; Roncero et al., 2020).

The importance of psychosocial support services in mental health recovery is increasingly being recognised. Like clinical services, psychosocial support services have had to adapt to deliver socially distanced services during COVID-19, a major adaptation being the introduction of telephone and teleconference-based support (referred to henceforth as tele-support). Governments have provided additional funding to support tele-support expansion to ensure continued access (Department of Health, 2020).

The evidence from clinical mental health and allied health services discussed above suggests that, despite potential barriers, psychosocial support services have the potential to be useful when delivered via technology. In fact, allied health research indicates that the use of technology may be particularly compatible with the kinds of supports routinely provided by psychosocial support services: interventions that aim to build individuals’ capacity through collaborative coaching approaches in natural environments (Ashburner et al., 2016). However, as noted, we could locate no research examining the use of tele-support to provide psychosocial support for people living with EMHCs. Nor has the impact of COVID-19 on people’s experiences of using community-based psychosocial support services been explored. While literature on telehealth in clinical mental health and allied health provide clues, differences may exist due to the different foci and common treatment modalities.

Understanding the impact of COVID-19 on psychosocial support service use will assist these services to: a) prepare for future waves and pandemics; b) adjust their services to an ever-changing ‘COVID-normal’ world; and c) consider how the adaptations necessitated by COVID-19 could be incorporated into a flexible suite of person-centred service delivery options in an ongoing way. A useful way of exploring the interaction between COVID-19 and psychosocial support is through direct lived experience insights from people who are living with EMHCs and using psychosocial support services. Service users’ subjective experiences are increasingly being recognised as critical to developing and delivering person-centred services (Thornicroft & Tansella, 2005). This paper examines service user perspectives to address the following research question: How did the COVID-19 pandemic shape people’s engagement in and experiences with psychosocial support services in Australia?

## Methods

### Study Design

This paper is a qualitative secondary analysis of data from written questionnaires and in-depth interviews conducted for a national evaluation of psychosocial support services. The research was conducted by a research team that included lived experience researchers, so a lived experience perspective informed the research at every step. Ethics approval was obtained for this study from the University of Sydney Human Research Ethics Committee (protocol # 2020/266).

### Context

Data were collected for this evaluation in Australia between June and October 2020. During this period and in the preceding four months COVID-19 was having a profound effect on the lives of Australians (Berger & Reupert, 2020; Duckett & Stobart, 2020; Edwards et al., 2020; Lupton, 2020; Rahman et al., 2020; Torales et al., 2020). From mid-March, lockdown restrictions were progressively implemented by federal and state governments. While rules varied by state, most Australians were required to stay at home except for purposes of getting food, attending to health or care needs, exercise and employment or education that could not be done from home. Visitors to private homes and public gatherings were prohibited and non-essential services closed. International borders were sealed, and movement was restricted between most states and territories. By May, the curve of new COVID-19 cases had flattened, and lockdown restrictions began to ease in most states. However, a ‘second wave’ of infection had commenced in the state of Victoria, prompting additional state border closures and severe lockdown measures in that state lasting for nearly four months.

### Sampling and Recruitment

Participants in the evaluation were currently or previously engaged with two federally funded psychosocial support programs – the National Psychosocial Support Measure (NPSM) and Continuity of Support (CoS). Questionnaires and Participant Information Sheets were distributed by all willing service providers of NPSM and CoS services across the country (n = 105) to all current and former program participants who they could contact. A total of 500 people completed the questionnaire. Participants for qualitative interviews were recruited via a final questionnaire item, which asked respondents if they were willing to discuss their experiences in an interview. A total of 349 people agreed and provided contact details. Maximum variation sampling (Palinkas et al., 2015; Patton, 2002) was conducted based on questionnaire responses to select an interview sample that included people with a range of demographic and service use characteristics. Efforts were made to contact 142 people. Sixty-seven were not able to be contacted (e.g., phone disconnected or did not respond to text or email messages). After written and verbal information was provided about the interviews, two people declined to participate further; 63 people provided informed consent and were interviewed.

### Data Collection

Questionnaire data collection was flexible to cater to respondents’ different needs. People could complete the questionnaire online, on paper (returned in a reply-paid envelope), or over the phone with one of the researchers. Questionnaire data were collected via REDCap, an online secure data capture tool with survey function. Where participants responded via paper questionnaires or telephone, responses were entered into REDCap by the researchers. Questionnaires included demographic items and questions about service involvement and satisfaction. They also included 3 open ended questions asking about “the best or most helpful thing” about the service, the “worst or least helpful thing”, and for any additional comments. While questionnaires did not specifically ask about COVID-19, it was often discussed in response to these open-ended questions.

In-depth, semi-structured interviews were conducted for a deeper and more detailed exploration of people’s experiences and perspectives (Rubin & Rubin, 2011). An interview guide containing open-ended questions was designed based on program aims and literature around psychosocial and recovery-based services. It was used flexibly to allow the interviewer to ask follow-up questions and explore the issues most relevant to each interviewee. As part of the interview, participants were asked whether the service had changed due to COVID-19 and, if so, how they had experienced these changes. Interviews were conducted by telephone or video-conference, lasting between 11 and 71 min (mean = 30 min). The range depended on how much each participant wanted to tell us, however even participants whose interviews were relatively short were able to share important information that contributed to the analysis. Most interviews were conducted by researchers who had their own lived experience of mental health issues. They were audio-recorded and transcribed verbatim for detailed analysis.

### Data Analysis

Responses to the 3 open ended questions in the questionnaire and all interview data were coded, extracting any data referring to COVID-19. These data were then analysed in detail using constant comparative analysis, a systematic and well-regarded qualitative analysis method (Charmaz, 2014; Glaser, 1978). Data were inductively coded line-by-line, with short names given to each concept apparent in the data to define the idea being expressed (Charmaz, 2014). Each new chunk of data was compared to previous data and existing codes to determine whether the underlying concepts were the same or different, and new codes were created as required. Similar codes were grouped together into higher level categories and relationships between codes were identified. This type of analysis ensured that concepts were grounded in the data rather than determined by pre-existing ideas (Charmaz, 2014). Involving researchers with lived experience of mental health issues in the analysis helped to ensure that it faithfully represented stakeholders’ views. NVivo’s case allocation and cross-tab functions were used to count the number of people who mentioned the identified issues. Authors have no known conflicts of interest and certify responsibility for the manuscript.

## Results

Demographic characteristics of people who provided data about COVID-19 are provided in Table 1. Of the 500 people who participated in the original study, a total of 121 participants discussed COVID-19: 59 in the questionnaire only, 55 in the interview only, and seven in both the questionnaire and interview. The latter are designated as ‘interview participants’ for the remainder of this paper, to avoid double counting.

**Table 1:** Program participants who discussed COVID-19

Characteristic	Questionnaire-only respondents		Interview participants	
	n%		n%	
Included participants	59	100%	62	100%
*Age*	(n = 57)		(n = 62)	
Range	22–74		22–69	
Mean (standard deviation)	47 (11.2)		47 (11.99)	
*Gender*	(n = 59)		(n = 62)	
Female	38	64.4%	34	54.8%
Male	19	32.2%	24	38.7%
Other	2	3.4%	4	6.5%
*Aboriginal or Torres Strait Islander origin*	(n = 58)		(n = 61)	
No	56	96.6%	54	88.5%
Yes	2	3.4%	7	11.5%
*Country of birth*	(n = 58)		(n = 59)	
Australia	45	77.6%	46	78.0%
Other	13	22.4%	13	22.0%
*First language*	(n = 58)		(n = 62)	
English	439	88.2%	56	90.3%
Other	59	11.8%	6	9.7%
*Area of residence*	(n = 59)		(n = 60)	
City/metropolitan area	43	72.9	44	73.3%
A regional centre	8	13.6	6	10.0%
A rural or remote area	8	13.6	10	16.7%
*Currently employed*	(n = 58)		(n = 62)	
Yes	5	8.6	7	11.3%
No	53	91.4	55	88.7%
*Diagnoses*	(n = 59)^a^		(n = 62)	
Schizophrenia spectrum and other psychotic disorders	11	18.6	9	14.5%
Bipolar and related disorders	12	20.3	9	14.5%
Depressive disorders	31	52.5	30	48.4%
Anxiety disorders	25	42.4	22	35.5%
Trauma- and stressor-related disorders	15	25.4	16	25.8%
Personality disorders	7	11.9	9	14.5%
Other	3	5.1	10	16.1%
*Length of involvement with service*	(n = 59)		(n = 59)	
Less than 1 month	2	3.4	2	3.4%
Between 1 and 3 months	12	20.3	7	11.9%
Between 4 and 6 months	10	16.9	11	18.6%
More than 6 months	35	59.3	39	66.1%
*Satisfaction with services on a scale of 1–10 (1 = “terrible – nothing good about it”; 10 = “fantastic – all I’d hoped for”*	(n = 59)		(n = 62)	
Range	3–10		2–10	
Mean (standard deviation)	7.0 (1.96)		7.5 ( 0.5)	

Notes: n = number of people who provided a response to each question

Percentages are calculated using the number of people who responded to each question rather than the total number

^a^Percentage total greater than 100 as people were able to provide multiple responses

COVID-19 was reported to have had a considerable impact, both on people’s lives and on their engagement with psychosocial support services. Issues relating to the person, their situation, and the actions of services interacted to influence their experiences and engagement with services in a COVID-19 environment. These impacts and interactions are described below. The numbers of participants who mentioned particular issues are noted, and participants are identified by participant numbers with ‘i’ denoting interview participants and ‘q’ denoting people who participated in questionnaires only.

### Impact of COVID-19 on Participants’ Lives

Although participants were not asked about the impact of COVID-19 on their well-being, 21 people (14i; 7q) described negative impacts. These related to the perception of risk of catching the virus, the impact of social distancing restrictions on their activities, uncertainty around the wider and future impact of the pandemic, and reduced access to formal and informal supports. For example, i22 explained that “all my health services stopped, so it was quite hard”, while i19 reported that “after a while, just sitting here day in and day out, I was feeling a bit not so good”. Others talked about feeling frustrated, sad, worried or anxious.

i17: I'd sit at home, this stupid COVID, and I'd cry and I just had myself so wound up that ‘we're never going to get over this, we're going to be stuck in lockdown’. I was always paranoid of when I had to go out in the street that someone was going to have that, and I was going to catch it.

### Impact of COVID-19 on Participants’ Engagement with Psychosocial Support Services

There was considerable variation amongst participants in terms of whether and how their engagement changed during the COVID-19 restrictions. Some reported that psychosocial support services had become more important and necessary than at other times or that their services had continued to support them. Other participants, however, reported reduced engagement. Most discussed how the mode of service delivery had changed.

### Engagement Became More Important (n = 9: 2i; 7q)

Due to the impact of COVID-19 on their lives, several people stated that their need for psychosocial support services had increased (n = 9; 2i; 7q). For example, q77 stated that the most helpful aspect of the service was:

q77: The contact - during COVID-19 it has been imperative to my mental health that I had contact from an external organisation to help me feel 'connected' to the outside world and to have someone to speak to about how I was feeling and what I was going through. Their contact has been so important and helpful to people in isolation and that having someone on the other end of the phone has made such a difference between coping and not coping.

### Continued Engagement (n = 21: 13i; 8q)

Eleven participants (3i; 8q), including 6 for whom engagement had become more important, reported that the psychosocial support service had helped them get through these difficult times, particularly by things like providing “a general catch up about how things are going” (q371) and letting people “know that there is someone looking out for me” (q366). Ten additional participants (10i) reported that the service had adapted well to the crisis, continuing to support them at similar levels throughout lockdown.

*i22:That was their strong point…* the agency tried really hard to make sure that people didn’t get isolated or didn’t lose their support.

### Engagement Reduced (n = 50: 29i; 21q)

For more people, however, engagement with the service was reduced due to closure of groups and activities, the inability to pursue goals that involved community participation, and reduced or delayed provision of individual supports.

#### Groups and Activities Reduced or Ceased

Thirty-two participants (21i; 11q) described their engagement with services decreasing because COVID-19 restrictions meant that group activities, such as support groups, outings and social gatherings could no longer happen and people could not attend the service in-person. This was a great loss to many participants, even though they understood that it was necessary. i13 explained: “it was more socially with clients or with staff, there was none of that for a whole two months and I just, it got to me.” q430 summed up the feeling by stating “I can’t wait for the lockdown to end and for all the other activities to start”. Once group activities restarted, access was often less than it had been previously because of limited group numbers (3i;1q).

#### Community-Related Goals on Hold

Eleven participants (10i; 1q) talked about how some of their goals for their work with the service had to be put ‘on hold’ because they involved interacting with other people in the community in a way that, during lockdown, was either impossible due to community venue closures or seen as too risky. Getting involved in community activities and making friends were the most common goals affected.

i20: One of my goals was to—like I really was wanting to get involved in like some group stuff, so outside of the house. That was all …it's been put on hold.

Other goals also stalled. For example, i21’s progress toward cleaning up her unit “just stopped” because her support worker could not be physically present, second-hand shops had stopped accepting donations and she could not find “a person who could take the rubbish away”.

#### Reduced or Delayed Individual Support

Twenty-seven participants (11i; 16q) reported other delays, disruptions, and lack of support which they attributed to COVID-19. For example, for q107, the ‘worst or least helpful’ aspect of the service was “COVID-19 restrictions preventing people to get help”, while for q191 it was “not enough support due to COVID”. Interview participants provided more detail, with some reporting that their contact with the services became less frequent or regular, reducing their access to social and emotional support. i17, for example, reported that, “a couple of times I got a couple of text messages from some of the staff. That probably only went for a couple of weeks and then they just became distant.” i42 shared a similar experience, saying that “when things were normal, I always had a regular day or time to see [my support worker], and then when COVID came, things became different.”

A couple of participants described promised support that had not gone forward even after restrictions were lifted and three participants (3i) expressed the opinion that services were maintaining stricter precautions than necessary, delaying getting back to pre-COVID support.

i8: [I would like] more social visits, I live alone so, I went to church yesterday and asked the pastor how many people he [can have] at his church worship and he can have to fifty. So, if you can have up to fifty, surely [my support worker] can come to my unit, just one-on-one.

### Mode of Engagement Changed (n = 75:47i;28q)

Seventy-five participants (47i; 28q) reported their psychosocial support service replacing at least some of their face-to-face sessions with telephone or internet-based communication. This involved: phone calls (n = 35; 26i;9q); one-to-one videoconferencing (n = 7; 5i;2q); and group videoconferencing (n = 10; 6i;4q), with some people describing services using multiple methods.

While four people (3i;1q) simply mentioned the change to tele-support without expressing an opinion, most told us how they experienced tele-support. Five participants (4i; 1q) reported finding advantages of tele-support over face-to-face sessions. Some found it convenient: “It's been really helpful that I don't have to leave my house and organise transport and stuff” (i24). For others it was a less threatening alternative.

i18: I'm occasionally not okay with face-to-face stuff. So it's been good for me to be having the video there because - I'm still seeing and being seen but it feels like it's one removed, so I'm not as intimidated.

Twenty more people (20i) indicated that they felt positive or neutral overall about having their interactions via tele-support, seeing it as a good replacement, or not making much difference to their experience. They said things like “I felt like the phone contact didn't take that much away from me” (i04) and “it’s like you’re there in real life” (i10).

However, 47 people (24i; 23q) indicated that the move to tele-support was negative for them. Eighteen questionnaire respondents answered the question about least helpful aspects of the service with comments like “no face-to-face contact during COVID-19” (q147). This was one of the most common “least helpful things” mentioned in questionnaires. Many interview participants also expressed a general preference for face-to-face interactions, stating that phone calls or teleconference “just hasn’t been the same”(i21) and “I prefer face-to-face, obviously” (i31). However, 21 people (15i;6q) provided more detail about problems they encountered.

Some stated that the physical presence of another person was important to them, especially in isolating times. i35 explained how tele-support “was a bit difficult for me. I found I wasn't as into it. I don't know why, but I just prefer the person-to-person contact… and just the physical connection”. With phone calls in particular, people missed being able to see the support worker and read their body language. Similarly, the support worker was thought to be less able to be sensitive to how the person was going.

*i60:[My support worker]* can seem to instinctively be able to find out what's going on with me. Just one look at me. You can't do that over the phone. *I could tell you the walls are pink here and you wouldn't know whether they're pink or blue or what.*

For these reasons, a couple of people expressed a preference for videoconferencing over phone calls. However, videoconferencing had its own issues, with a couple of people reporting difficulty connecting, interruptions, and a reduction in meaningful interaction.

q36: Communication from others in the group was stilted. The group participants were difficult to see and sometimes hard to hear…The online group was nothing like the cohesive and worthwhile groups I have done in person.

Some reported that using phone or videoconferencing: made it more difficult to develop trust and rapport with their support worker, reduced their ability to concentrate, and made them feel less relaxed and comfortable, impacting on the therapeutic value of the interaction. For example, i40 said that “it sort of almost feels like you're talking to a machine”, while i32 described becoming bored quickly when interacting through the computer and that “a lot of times I just want to hang up.”

Most people accepted tele-support as being necessary, even if not ideal. For example, i5 explained that “for me it's just another challenge… something I've got to get through until we're back on our feet and mobile again”. Four participants, however, (3i;1q) reported having reduced their engagement with the services because of the perceived insufficiency and difficulty of tele-support. i19 explained that, because the group was online “I didn't want to do all that, so I just left it.”

Twenty participants (20i) reported that they had continued to have at least some face-to-face interactions with their support worker during the COVID lockdown. People described modifications, such as sitting in the back of the car, wearing a mask, answering questions beforehand about any symptoms, and interacting outdoors at a distance. People tended to appreciate this continued face-to-face support.

i36:[My support worker] was well aware of the fact that meeting her in person, like physically seeing someone and being around other people once a week was very important to my mental health… we'd go and sit down at a park and just sit apart from each other and talk.

#### Factors Affecting the Impact of COVID-19 on Service Engagement and Well-Being

People described several factors that they felt influenced the impact of COVID-19 on their wellbeing and their engagement with services. These included: their location, living situation and pre-COVID lifestyles; any physical health conditions; their access to and comfort with technology; how the support worker facilitated technology use; their pre-COVID relationship with the support worker; and communication from the organisation.

Where people lived affected the level and duration of restrictions and the perceived risk of COVID. For example, those living in Victoria were experiencing a second extended lockdown, while in other states, severe restrictions had been relatively short-lived. Several people (n = 6i) mentioned that living alone had heightened the impact of the restrictions. For example, i13 explained that “it was hard, all you'd do is go to work, come home—it really hurts your life”. A couple of people were grateful to have a flatmate, caring neighbour or a pet to provide contact during lockdown. For some, particular features of their situations, such as living in a remote area, having few social contacts, or having family members in different states increased their feelings of isolation. This sometimes made psychosocial support more important. However, others (n = 6i) noted that the impact of COVID-19 on them was not as severe because “I’m not a very social person” (i7), or “I was isolating way before COVID came in so nothing's really changed in my life in that respect” (i41). People felt the effects of COVID on services more if they were previously involved with and highly valued the group activities offered, which were no longer available.

Participants who had a physical condition that increased vulnerability to COVID-19 or who cared for someone who did (n = 5i) reported increased restrictions and worry, as exemplified by i33, who had a chronic health condition.

*i33: I try to be really careful and I guess that’s stressful in itself. Then when you go out, there’s all the stuff you’ve got to remember and things you can do and you can’t do, and meant to do, and yeah, so it has put an extra bit of stress on.*

Comfort and familiarity with using technology was very important for people engaging with tele-support. For example, i31 explained how “I've done work with psychologists and psychiatrists and stuff through Zoom, so I'm pretty used to it now”. On the other hand, i38 stated that “I’m not very comfortable at all online with anything.” People also needed to have the right equipment and services. Some people who did not have a computer, for example, or who had poor internet reception, felt more isolated or that they faced additional barriers to a smooth and positive experience of tele-support.

How the technology was used and facilitated by the service providers was also important. While two participants mentioned that staff needed more training, time and funding to transition to online service delivery, others talked about how teleconferencing was “made user-friendly by the staff” (i9) or how staff persevered in helping them to learn the technology. One participant was impressed with the support worker’s use of videoconferencing features.

i18: We can type down little notes of things that we're interested in and we can both see them and talk about them… then at the end of the call we both get a copy of that.

Six people (4i; 2q) mentioned that their comfort in engaging with their support worker remotely was influenced by their existing relationship. Five had not previously met their support worker face-to-face or had only met once, and they found this difficult. For example, q451 said she could not trust her support worker because they had never met face-to-face. On the other hand, i56 described how “I have already met with them on face-to-face meetings so changing to telephone conversation did not make any significant change.”

Eight people (7i;1q) indicated that their experience was influenced by the communication between themselves and the service, which determined their knowledge and understanding of what was going on and what to expect. For example, q265 said that they were “not really getting much contact lately, and not knowing what's going on with it.” i40, on the other hand, felt that the communication had been good.

*i*40: *They made it very clear. It was very obvious what was happening, but they explained it to me that ‘your program – it's just been delayed, it hasn't stopped, you haven't missed out, but because of COVID and the COVID rules and lockdown’*.

Twenty-four participants (20i; 4q) described support workers proactively engaging with them throughout COVID, by making contact and checking in with them, for example, through increased text messaging. This was generally appreciated.

i17: Through this Coronavirus, it's been a really bad time, my support worker has contacted me consistently and if she isn't there she will get someone else to contact me.

Five participants felt reassured that they could call the service when they needed to, with i48 saying that she “always knew I had phone access to [my support worker] when I needed it.” On the other hand, i42 explained that, despite needing support during lockdown, she had not phoned her support worker because “I didn't realise that it's okay to.”

## Discussion

Psychosocial support programs provide an essential service that addresses people’s basic needs to better manage their mental health, stay connected and actively work on their recovery goals. Physical distancing requirements associated with the COVID-19 pandemic have impacted psychosocial support service delivery (Productivity Commission, 2019). This paper is the first to examine how the changes associated with a pandemic shape people’s engagement in and experiences with these services.

Our findings indicate that continued engagement with psychosocial support services is important to service users. Yet our participants were more than twice as likely to report reduced engagement than continued engagement at a similar level. While some activities, such as attending community events, may have needed to be put on hold, other delays and disruptions were not an inevitable consequence of social distancing. For example, some participants received socially distanced face-to-face services and others attended online groups, even involving practical activities like cooking. Reductions in individual contacts, an important source of social and emotional support, were likely due to upheavals caused by services being overwhelmed, which can largely be attributed to the speed at which they were required to adapt and change modes of service delivery in a COVID-19 environment. Our data suggests that a lack of familiarity with tele-support options amongst both service providers and users may also be important. This has highlighted both a need to prepare for a more agile service response in future situations and an opportunity to incorporate new modes and options into standard service provision.

Using technology to deliver health services has, for many years, been advocated as a viable way of facilitating access for those who face barriers, particularly for those living in rural and remote areas (Dew et al., 2012). It is increasingly proposed as a promising model to increase accessibility to specialist services within other areas because of its many benefits (Dew et al., 2012; Madigan et al., 2020; Nadeem et al., 2020). Yet telehealth and tele-support have not been widely practiced for people with EMHC and have, until recently, been poorly supported by funding structures in Australia and elsewhere (Snoswell et al., 2020). Perhaps a silver-lining on the cloud of COVID-19 has been that it has necessitated the adoption of this potentially beneficial mode of service and the upskilling of providers and users.

Encouragingly, in our study, around a third of participants who reported receiving some psychosocial tele-support services were positive or neutral in their perceptions of it as a replacement for face-to-face services. Attending to the barriers described by the other two thirds of participants will be critical to facilitating increased comfort and uptake of tele-support services, both in the event of further pandemic events and to increase flexibility and choice in normal service delivery.

Issues with technology amongst people with EMHC are often cited as a barrier to telehealth. For example, the Productivity Commission (2019) reported that many experienced difficulties with videoconferencing facilities due to a lack of access to suitable equipment, low technical literacy, inability to afford access to large data packages and unstable internet connection. This is likely to be the reason that most tele-support services reported by our participants were via telephone. However, issues around not being able to see the support worker were reported as being important to many people. Interestingly, the available research evidence indicates that therapeutic relationships are not comprised by either telephone or video-conferencing modes of service delivery when compared to in-person psychological therapy (Irvine et al., 2020). Although certain challenges have been described related to telephone-based provision of psychological therapy, the greater sense of control and invisibility afforded by this mode of service delivery may also support people to feel more comfortable and less inhibited, increasing self-disclosure (Davidson & Harrison, 2020). Regardless, any perceptions of the inferiority of tele-support would need to be addressed in order to support its uptake and effectiveness for service users. For instance, setting users up to confidently access teleconferencing, through hands on training and resource provision, would take time and money but, given the potential savings in terms of support workers’ travel time, these steps to increase the acceptability of tele-support to service users (Hines et al., 2019) may be a viable investment. In addition, helping service users to develop skills in technology can facilitate other sources of support, especially during a pandemic situation, such as maintaining social connections and doing online shopping (Winstanley et al., 2020).

Our study indicated that familiarity with technology and tele-support was important to people’s experiences. This is supported by research in allied health, which has suggested that the acceptability of telehealth services increases with experience and exposure (Hines et al., 2019; Hines et al., 2015; Rietdijk et al., 2020). While difficult in the midst of pandemic restrictions, taking time to introduce service users to tele-support as an option is likely to increase their comfort with the medium. Further, research suggests that using telehealth in conjunction with face-to-face support is likely to be more viable and acceptable than offering telehealth only (Santesteban-Echarri et al., 2020; Topooco et al., 2017). Increased capacity for and comfort with tele-support in ‘normal’ times will reduce the upheaval involved when and if it becomes a necessity.

However, working with service users to facilitate access to and comfort with tele-support requires support workers to have positive attitudes and skills around tele-support. Health professionals’ attitudes have been identified in both mental health and allied health research as a barrier to telehealth implementation (Topooco et al., 2017; Wind et al., 2020). In fact, a number of studies have indicated that service users are more likely to have positive attitudes towards telehealth and be more willing to engage with it than health professionals are (Graham et al., 2020; Iacono et al., 2016). Health professionals express concerns ranging from the impact on therapeutic relationships, the adequacy of technology, the willingness or capability of service users to engage in telehealth services, and its appropriateness for ‘hands on’ interventions (Johnson et al., 2021; May & Erickson, 2014). Again, attitudes tend to become more positive with increased experience (Hines et al., , 2015, 2019).

Specific skills and strategies are likely to be needed to optimise tele-support experiences for service users. For example, psychologists using technology for appointments during the pandemic reported that they learned strategies such as using words more to communicate empathy and ‘check in’ with clients, and using more exaggerated physical gestures (Downing & Harriott, 2020). Our research suggests that psychosocial support workers, like health professionals, may benefit from being trained to: manage technical aspects of tele-support; develop a ‘webside manner’; adjust their practice to facilitate building therapeutic relationships; and foster the comfort, confidence and skills of service users around tele-support (Moreno et al., 2020; Stefana et al., 2020; Torous et al., 2020). Such training and guidelines for practice would ideally be evidence based and developed through co-design processes with service users to produce appropriate, effective tools (Moreno et al., 2020). Mpango (2020) emphasised that further research, involving services users at the highest levels, is needed to understand how alternatives to face-to-face delivery of psychosocial interventions may best be implemented to maintain human connection and continue to develop working relationships.

Several limitations to this study should be noted. First, service users who had lost contact with services, whether due to negative experiences or other reasons, are not represented in this study. Second, the same ‘tools’ of engagement were used in interviews as in tele-support – telephone and video conferencing. It is likely, therefore, that those with the greatest barriers to tele-support options may not have participated in the interview component of this study. Lastly, as a secondary analysis, the impact of COVID-19 was not the primary focus of the study. There were no explicit questions in the questionnaire and only one in the interview guide about the impact of COVID-19 so the relevant data from each participant was limited. Further, sampling for the original study was not based on saturation of COVID-19 related categories. However, the spontaneous comments around COVID-19 throughout questionnaires and interviews indicate that it was an important issue, and the number of people’s perspectives represented in the analysis enabled coverage of a considerable range of issues.

## Conclusions

Psychosocial support services have been forced to engage with tele-support throughout COVID-19. While this has not always been a positive experience for service users, it has opened up possibilities for the future. Some people with EMHCs may never find tele-support practical or acceptable, and person-centred services need to acknowledge and respect that. However, our research shows that others are, or may well be open to tele-support if given the right resources and opportunities to learn and use it in positive circumstances. Given the potential benefits of tele-support and the learnings from COVID-19, rather than abandoning tele-support in a post-pandemic world, psychosocial support services, their staff and clients would benefit from additional investment in a planned and well thought out transition toward blended service delivery where this is compatible with person-centred practice. This requires research, evidence-based guidelines that are co-produced with people with EMHC around tele-support practice, and government support through ongoing funding and policies to move into this new era of healthcare service provision wherein tele-support is offered as part of a suite of service delivery options.

## Data Availability

Data is potentially identifiable, therefore cannot be made freely available.
